# Effect of Charcoal on the Quality of Vermicompost Produced With Water Hyacinth and Cow Manure

**DOI:** 10.1155/tswj/1086347

**Published:** 2025-03-24

**Authors:** Solomon Girmay Berhe, Ali Seid, Berhanu Abraha Tsegay, Shinjiro Sato, Getahun Yemata Lule

**Affiliations:** ^1^Department of Biology, Science College, Bahir Dar University, Bahir Dar, Ethiopia; ^2^College of Agriculture and Environmental Science, Bahir Dar University, Bahir Dar, Ethiopia; ^3^Department of Biology, College of Natural and Computational Science, Raya University, Maichew, Ethiopia; ^4^Department of Science & Engineering for Sustainable Innovation, Soka University, Soka, Japan

**Keywords:** biofertilizer, composting, eutrophication, lettuce, utilization, weed

## Abstract

The significance of water hyacinth (*Pontederia crassipes* (C. Mart.) Solms (Pontederiaceae) vermicomposting lies in its ability to effectively manage its invasiveness while also improving soil fertility and supporting sustainable agricultural practices. This study evaluates the effects of charcoal addition on the composting followed by the vermicomposting of water hyacinth (WH) and cow manure, focusing on the growth, reproduction, and survival of *Eisenia fetida* earthworms and the quality of the resulting vermicompost. The treatments included WH and cow manure (2:1) with 7.5% charcoal (T1), 5% charcoal (T2), and without charcoal (T3). Composting (30 days) followed by vermicomposting (60 days) was conducted in a controlled greenhouse environment. Samples were collected biweekly to analyze temperature, moisture, pH, electrical conductivity, total nitrogen, total available phosphorous, and total potassium contents, vermicompost yield, and earthworm growth parameters throughout the process. Results showed that T1 and T2 significantly improved earthworm weight (8.23–16.0%), number of earthworms (65.0–69.0%), cocoon production (37.0–49.0%), and hatchling count (49.0–77.6%), compared to T3 (control). Also, T1 and T2 increased pH (4.30–5.75%), total nitrogen (53.6–62.5%), total available phosphorus (61.6–117%), and total potassium (47.5–71.3%) and reduced electrical conductivity (9.91%), total organic carbon (17.9–42.1%), carbon: nitrogen ratio (46.5–64.4%), and vermicompost mass (22.0–28.3%) at the end of the vermicomposting period, compared to T3. Moreover, T1 and T2 exhibited significantly higher lettuce seed germination (92.4–93.5%), germination index (76.2–80.4%), shoot elongation (46.0–92.5%), and root elongation (9.00–15.0%), compared to T3. However, in most of the results, there was no significant difference between T1 and T2. This finding suggests that the use of T2 (5.00%) charcoal addition enhances vermicompost quality to optimal maturity and stability.

## 1. Introduction

Water hyacinth (*Pontederia crassipes* (C. Mart.) Solms (Pontederiaceae), formerly Eichhornia crassipes) is recognized as one of the most difficult macrophyte weeds globally, causing serious ecological problems in aquatic ecosystems globally [1]. Water hyacinth (WH) is a free-floating aquatic weed that can reach heights of up to 65 cm. It can reproduce both sexually and asexually, though dominated by the vegetative growth. A single WH plant produces up to 3,000 seeds that remain dormant for several years. According to Albano Pérez et al. [2], there are 400–3,400 seeds per square meter of vegetation [2].

Controlling its spread is difficult due to its unique, versatile growth ability to double biomass in five days, reaching up to 17.5 tons of dry biomass per hectare each day [3]. Its allelopathic characteristics make it even more difficult to control, which calls for efficient management techniques [4, 5]. Its broad root system and capacity to adapt to different conditions allow it to effectively absorb nutrients and promote quick growth and reproduction [6]. In addition, its nutrient loading in aquatic systems affects land degradation and inadequate waste management techniques, which results in eutrophication in freshwater lakes and rivers [7]. WH has thus severely affected Lake Tana, in Ethiopia, invading as high as 2,126.9 and 592.4 hectares of the lake, producing between 1,990,787.8 and 554,523.88 tons of dry biomass annually [7]. WH invasion severely disrupts the lake's ecosystem services, affecting biodiversity, irrigation, and fishing activities, while also limiting water accessibility and increasing evaporation [8].

Efforts to manage WH mostly through physical removal, with excavators and boats, have been mainly unsuccessful in Ethiopia, especially around Lake Tana [9]. The WH biomass is dumped on the lakeside or agricultural fields or burnt openly, leading to hazardous air pollution and contamination of lake waters, which poses public health risks such as increased malaria [10] and lake eutrophication [11]. Therefore, cost-effective technologies for the safe disposal of WH biomass are essential, allowing its conversion into a soil conditioner and nutrient source for sustainable agriculture.

The high organic matter and essential nutrients of WH (nitrogen 2.8-32.9 g/kg and phosphorus content 4.32-8.20 g/kg [12, 13] make it the best candidate as a source of bio-fertilizer and soil conditioner [12–14]. However, fresh WH cannot be directly used as a soil amendment due to seed germination risks [2, 15] that may lead to further weed proliferation, high moisture content (94.16%) [16] that can lead to an anaerobic environment, high cellulose (23-57%), hemicellulose (33%), and lignin (10%) contents that resist microbial breakdown [17]. Moreover, WH accumulates toxins and allelopathic compounds, such as loliolide, that inhibit plant growth [4, 5]. To help manage these WH problems, it can be combined with locally available and low-cost amendments such as cow manure and charcoal that undergo an anaerobic pre-composting process. This approach reduces earthworm mortality, eliminates pathogens, and softens the compost mixture [18]. Following this, ventilation and stabilization can lower moisture content and further eliminate pathogens [19]. Pre-composting is indispensable for making substrates suitable for worm activity, thus reducing mortality and improving the compost mixture's quality [19, 20]. Pre-composting eliminates pathogens, softens substrate mixture, and makes substrates suitable for worm activity, thus reducing mortality and improving the compost mixture's quality [19].

Composting and vermicomposting are emerging cost-effective biological methods for converting biological waste into valuable biofertilizer [21]. Composting involves the breakdown of waste matter by bacteria, fungi, and actinomycetes, transforming it into a sanitized, stable, and matured organic material with enhanced biological, chemical, and physical properties [22]. This sanitization occurs during the initial aerobic phase of composting, when temperatures rise to 45–70°C [18, 23]. The elevated temperatures create an environment that kills many pathogens, effectively reducing potential health risks associated with composting wastes [20, 22]. The vermicomposting process involves the collective decomposition of earthworms and microorganisms, which is becoming increasingly popular due to its versatility and transforming it into nutrient-rich castings that serve as a valuable soil amendment [20, 22].

Compared to composting, vermicomposting often yields superior results, with higher nutrient availability and organic matter retention [22, 24]. Vermicompost offers a gradual release of nutrients, humic substances, and stable organic compounds and stimulates plant development by raising plant hormone levels [19, 22, 24]. Also, vermicomposting adds growth-promoting chemicals, micro- and macronutrients, and beneficial microbes to soil, which are important for soil health and sustainable agriculture [25]. Furthermore, the economic benefit of vermicomposting exceeds that of composting, and because selling earthworms could provide income, the practice's economic viability is also encouraging [19, 22, 24].

The current vermicomposting of WH that uses the sole use of cow manure as a bulking agent extends time for maturity and stability, limiting its practicality for subsistence farmers [26, 27]. For instance, vermicomposting WH with cow dung alone can take up to 110 days [27]. Consequently, optimization techniques are important to encourage farmers to adopt this technology and mitigate the ecological impacts of WH on fresh water aquatic ecosystems [28]. This study aims to combine WH with cow manure enriched with *Acacia mearensii* charcoal to assess the quality of the resulting compost. Vermicomposting enrichment involves the addition of bulking agent materials that are rich in nutrients and microbes or create enabling conditions to enhance the vermicomposting process, resulting in a higher fertilizer value of the end product [22, 24]. Recent studies suggest that utilizing cow manure with biochar or charcoal can effectively enrich the end product of vermicomposting earthworms [19, 22, 24].

In this study, cow manure and charcoal, low-cost amendment materials, were selected due to their availability and reported benefits in enhancing vermicomposting. Cow manure provides microbes, essential food for both microorganisms and earthworms, that improve the decomposition process [18, 22]. Charcoal provides habitat for microbes, adsorbs heavy metals and toxic substances [29], balances the C/N ratio [30], enhances porosity, facilitates aeration [31], provides habitat, and maintains optimum moisture for microbial and earthworm activity [32] and enhances nutrient retention [19]. The combination of these materials enhances the compost structure, stabilizes pH, enriches the final product, and benefits soil health and plant growth [23]. However, it is essential to manage the application of charcoal carefully, since excessive amounts can lead to nutrient imbalances [26]. The high C/N ratio of WH can influence the composting process; a C/N ratio greater than 30 may alter microbial activity and prolong composting time, while a lower ratio can lead to nitrogen loss in the form of ammonia (NH_3_). For effective composting and vermicomposting, the ideal C/N ratio of the feedstock mix should be between 20 and 30 [19, 22, 33]. Microorganisms in composting utilize carbon as an energy source and nitrogen for cellular structure. As these organisms consume carbon approximately 30 times faster than nitrogen, a C/N ratio closer to 30 is optimal. High C/N materials, such as charcoal (C/N =97.9), can enhance the vermicomposting of WH (C/N =42.7) and cow manure (C/N =28.4) [30]. Given the huge potential benefits of WH, there is a lack of standardized methodologies for producing vermicompost from WH and cow manure with the addition of charcoal. The objective of this study was to produce a high-quality vermicompost from WH after incorporating it with cow manure and charcoal. The specific objectives of this research were: 1) to investigate the effects of cow manure and charcoal amendments on the vermicomposting of WH using *Eisenia fetida,* and 2) to evaluate the quality of the resulting vermicompost as a bio-fertilizer. This research addresses the urgent need for improved WH management strategies, leveraging locally available, low-cost amendments to optimize the vermicomposting process. By converting this significant environmental challenge into an agricultural opportunity, the study aims to contribute to sustainable agricultural practices and enhance soil fertility in affected regions.

## 2. Materials and Methods

### 2.1. Substrate Collection, Preparation, and Experimental Set-up

Fresh WH and cow manure were collected from the Abay River (11^0^33'27.78”N. and 37^0^23'59.93” E.) and Bahir Dar town (11^0^35'15.91” N. and 37^0^24'07.55” E.), respectively, during the dry season. The WH was sun-dried for 1 week, chopped manually, and sieved (<2 cm) [21]. Also, locally produced *Acacia mearensii* charcoal was collected from the local market and then manually crushed and passed through a 2 mm sieve. *E. fetida* worms used for the vermicomposting process were collected from Weramit, Amhara Research Center, Bahir Dar, Ethiopia. Prior to adding to the composting bins, the sieved WH (12 kg) and cow manure (6 kg, 50% dry weight basis) were thoroughly mixed with powdered charcoal (<2 mm) at 0, 5, and 7.5% (w/w) as shown in Table 1. The WH: Cow manure mixture was adopted after Bernal et al. [27]. Also, the WH:Cow manure: Charcoal mixture was adopted after the recommendations of Jain et al. [34], who recommended effective WH composting needs recalcitrant material for better degradation. Then, in order to optimize the product in the shortest possible time, the mixtures were aerobically composted [35] for 30 days in a smooth rectangular cemented bin (1m length, 0.5m width, 0.5m height) (Figure S1).

Then, after the 30-day pre-composting period, the mixtures in each bin were thoroughly turned and ventilated manually for two weeks in order to provide suitable aeration to the earthworms [18, 27]. On the same day, the vermicomposting process started with the introduction of acclimatized adult *E. fetida* worms (total average weight: 775 g, 864 numbers bin^−1^), which were managed for the next 60 days. The earthworm's weight was determined based on the premise that earthworms can eat half of their body weight per day so that the vermicomposting process will be accomplished in the shortest possible time [36]. The fine, smooth cement bins (Figure S1) were kept under 12/12 h light/day temperature (20–25°C) and moisture content (MC) 60–70% by periodic sprinkling of fresh tap water as described by Gusain and Suthar [37]. All bins were covered with jute sheets to prevent direct sunlight, create a dark environment, and protect against predators like pests, frogs, birds, and wind [38]. The experiment was arranged in a completely randomized design (CRD) where three replicates were run for each treatment (n =3).

### 2.2. Sampling, Monitoring, and Analyses

#### 2.2.1. Sampling and Monitoring

**Figure 1:**
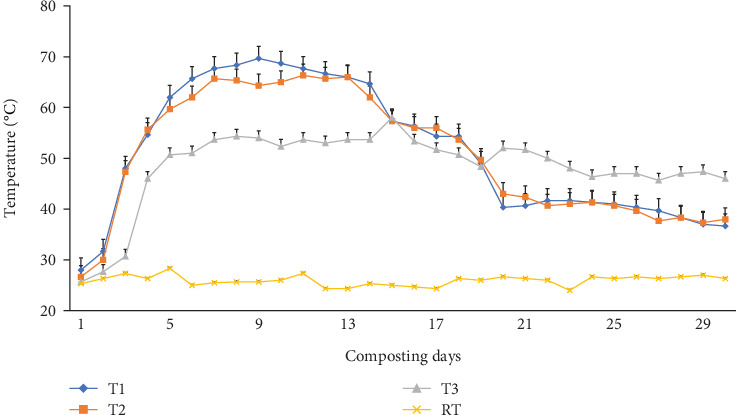
The mean temperature changes (°C) in the composting bins during the first 30 days. RT = room temperature; T1 = WH + cow manure (2 : 1) + 7.5%charcoal; T2 = WH + cow manure (2 : 1) + 5%charcoal; T3 = WH + cow manure (2 : 1).

Two hundred grams (g) of fresh homogenized composite samples free from worms, hatchlings, and cocoons were drawn in triplicate from five points (1 from each corner and 1 from the center) at 0, 30, 45, 60, 75, and 90 days from each bin for the physicochemical analysis [25, 39]. Day 0 refers to the initial date when the WH was co-mixed with cow manure and charcoal; day 30 refers to the composting day and when simultaneously earthworms were inoculated; and day 90 refers to the final vermicomposting date. Midday composting temperatures were recorded in the middle layers of composting mixtures in all bins, similar to soil sampling to a depth of 0-10 cm using a 30 cm-long mercury thermometer, on a daily basis during the 30-day composting period and then every 15 days until the end of the vermicomposting day. Also, mid-day greenhouse temperature and humidity were measured using a thermometer and hygrometer, respectively. The vermicomposting process was held inside a greenhouse facility in order to avoid disturbance, mainly due to rain, and create a successful decomposition process. The greenhouse facility has a uniform opening at the bottom for ventilation (50 cm wide along its whole perimeter) and experienced an average mid-day temperature of 25°C (Figure 1).

Air-dried samples were stored at 4°C for the determination of total nitrogen (TN), total available phosphorous (TAP), total potassium (TK), pH, and electrical conductivity (EC) [25, 39–41] or oven-dried at 105°C for 24 h for the determination of MC, total organic carbon (TOC), volatile solids, and ash content [42, 43]. TN and TAP were determined after a preliminary digesting of a 0.1 g sample treated with 98% (v/v) sulfuric acid and 30% (v/v) hydrogen peroxide. TN was measured based on the Kjeldahl method, and TAP was measured using the anti-Mo-Sb spectrophotometry method in a UV spectrophotometer (UV-1800, Shimadzu, Japan) as described by Gong et al. [44]. TK was determined by flame photometry after digesting a 0.2 g air-dried, 0.2 mm sieved sample with 10 mL of H_2_SO_4_ and HClO_4_ (5:1) at 300°C for 2 hours. The carbon to nitrogen (C/N) ratio was calculated from the values of total organic carbon and nitrogen content [45]. The pH and EC were measured using a calibrated pH meter and EC with a conductivity meter, respectively, after mixing 3 g of dried samples with 30 mL of distilled water in a 1:10 (w/v) ratio as indicated in [39–41]. This mixture was then mechanically shaken for 2 hours, incubated, and then the extract was filtered for analysis [46]. TOC was determined by measuring the weight loss upon ignition at 550°C in a muffle furnace, which indicates the amount of VS as shown in Equations (2) and (3) below [1, 46]. The MC of the samples was calculated after a 100-gram fresh sample was dried for 24 hours at 105°C on weighted crucible plates [47]. The ash content was determined after the oven-dried samples were ground, and 5 g of this sample was ignited at 550°C for 2 hours in the muffle furnace [46]. Calculations of MC, ash, volatile solids, and TOC samples are given in Equations (1)–(3).

(1) $$\begin{array}{c} \text{Moisture content }\left(\%\right)=\frac{\text{ Fresh sample}-\text{Sample oven dry weight}}{\text{Fresh sample }}*100 \end{array}$$

(2) $$\begin{array}{c} \text{Ash content }\left(\text{g kg}-1\right)=\frac{\text{ Initial weight}-\text{weight after burning}}{\text{Initial weight }} \end{array}$$

(3) $$\begin{array}{c} \text{TOC }\left(\text{g kg}-1\right)=\frac{\text{Volatile solids}}{1.8}=\frac{100-\text{Ash}\left(\%\right)}{1.8} \end{array}$$

#### 2.2.2. Earthworm Biological Analyses

Earthworm average weight, number of cocoons, and hatchlings were observed and recorded during the 60-90th days from the initial day of composting, while individual worm biomass and total worm weight gain were measured during the 30^th^–90^th^ days prior to watering every 15 days, as described in [39–41]. The earthworms, cocoons, and hatchlings were separated from the substrate manually, cleaned in fresh tap water to remove adhered impure materials from the body, and then weighed on a live weight basis. Finally, all the earthworm cocoons and hatchlings were returned to their respective containers. Based on the obtained data, growth parameters such as weight gain and growth rate (mg/worm/day) were calculated. The fresh worm biomass was recorded using a weight balance (Citizen Balance, Model-CG203) and determined after subtracting the initial biomass from the final biomass. The earthworm growth rate was calculated as shown in Equation (4) by Ferraz Ramos et al. [48].

(4) $$\begin{array}{c} \text{Earth worm growth rate}=\frac{\text{Xtx}-\text{Xti }}{\text{Ntx}}\; \end{array}$$

Where: *Xtx* is the average biomass (mg) in ‘x' time, *Xti* is the average initial biomass (mg), and *Ntx* is the time (days) from the beginning of the vermicomposting process.

#### 2.2.3. Vermicompost Productivity

The VC produced was weighed after the worms, cocoons, and hatchlings were hand-sorted, dried under shade (15% MC), and then weighed in kilograms. The VC productivity was calculated in percentage as described by Ramnarain et al. [49], as shown in Equation (5).

(5) $$\begin{array}{c} \text{VC mass change }\left(\%\right)=\frac{\text{ Harvested VC }\left(\text{kg}\right)\;}{\text{Total mass of feed}\left(\text{kg}\right)}*100 \end{array}$$

### 2.3. Seed Germination Assay

The vermicompost maturity and toxicity assay through seed germination is the simplest and most used biological method [25, 39, 50]. Thus, the vermicompost extracts approach, which was reported as appropriate for its higher germination response than direct sowing into soil or vermicompost soil mixes [40], was used in this experiment's seed germination test. Thus, separate VC samples were taken from T1, T2, and T3 bins at the end of 90 days for the phytotoxicity test. The VC extract was prepared according to the methods shown by Rupani et al. [50] from 100 g of VC (15% dry weight) and soaked in 200 mL of distilled water for 24 h and thoroughly shaken for 6 h. The content was centrifuged at 8000 rpm for 20 min at 20°C and then filtered through a Whatman No. 4 paper. This was considered a 100% extract. Further dilutions were made by adding distilled water to obtain 0, 25, 50, and 75% (v/v) extract treatments [50]. Lettuce seeds were used as a bio test [51]. Twenty-five seeds were placed on filter paper lined on the plate and then treated with 5 mL of VC extract or 5 mL of distilled water (control). Treatments were arranged in a complete randomized design (CRD) inside the greenhouse at 25°C for 3 days in three replications. After 3 days, the numbers of seeds germinating in each experimental plate were counted, and root lengths were measured. In order to assess the inhibitory impact on root development and germination index (GI), aqueous VC extracts were utilized [40]. GI is widely used to assess vermicompost maturity and is computed as the product of viable seed percentage, determined by monitoring seedling emergence, the number of germinated seeds, and root length growth [50] 3 days after sowing (DAS) as shown in the following Equations (6) and (7).

(6) $$\begin{array}{c} \text{GI}=\frac{\text{Next }}{\text{Nctrl }}*\frac{\text{RLext }}{\text{RLwater }}*100 \end{array}$$

(7) $$\begin{array}{c} \text{Root elongation}=\frac{\text{ RLext }}{\text{RLwater }}*100 \end{array}$$

Where N_ext_, N_ctrl_ represents the number of germinated seeds in the extracts and distilled water, respectively, and RL_ext_, RL_water_ show the root length in the extracts and distilled water, respectively.

### 2.4. Statistical Analysis

A one-way analysis of variance (ANOVA) was used to test the significant differences among treatment means in the production and characterization of compost and effects on earthworm biology-related factors, and the least significant difference (LSD) test was used to compare the treatment means at 5%. Also, a separate two-way ANOVA was made to analyze the statistical significance of the difference among the vermicompost extract types and extract concentrations on lettuce seed phytotoxicity test treatments. Turkey's honestly significant difference (HSD) test at the p <0.05 level was also performed to identify the difference between the means of the GI and GP measurements. HSD was used because it helped pool more than four treatments and compare with the control group [52]. The SAS statistical package (9.4 version) was used to analyze the data.

## 3. Results

### 3.1. Raw Material Physicochemical Characteristics

The physico-chemical characteristics of WH, cow manure, and charcoal before composting are shown in Table 2. The pH of WH, cow manure, and charcoal was found to be alkaline. The EC of cow manure was significantly (p <0.05) greater than that of WH and charcoal. The TN content in the cow manure was considerably higher than that of WH and charcoal, which indicated that cow manure could be helpful in reducing the C/N ratio of the WH compost.

### 3.2. Changes in Temperature, Moisture, pH, EC, and TOC

**Figure 2:**
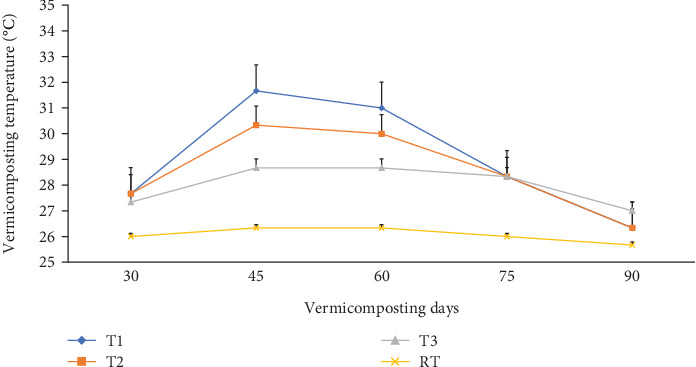
The mean temperature changes (°C) during the vermicomposting process of WH biomass. RT = room temperature; T1 = WH + cow manure (2 : 1) + 7.5%charcoal; T2 = WH + cow manure (2 : 1) + 5%charcoal; T3 = WH + cow manure (2 : 1).

Temperature plays an indispensable role in the earthworms' growth and development. The temperature varied in all bins significantly (p <0.05) during the 30-day composting (Figure 1) and 60-day vermicomposting period (Figure 2). Charcoal additions to the composting materials had a significant (p <0.05) effect on the temperature variation of these mixtures. A thermophilic temperature (above 45°C) was observed after the 3^rd^ day in T1 and T2. On the other hand, the temperature in T3 (control) reached around 46°C after the 4^th^ day. Charcoal addition to the composting mixture (T1 and T2) made the thermophilic temperature rise above 45.0°C, which lasted for 17 days (3^rd^–19^th^). The maximum temperature was observed after 9, 11, and 15 days after the start of the composting process in T1 (69.6°C), T2 (66.3°C), and T3 (58.0°C), respectively (Figure 1). But it continued till the end of the composting days (4^th^–30^th^) in the control treatment (T3). Between days 20 and 30 of the composting process, a significantly (p <0.05) declining temperature to mesophilic temperatures (36.6-43.3°C) was observed in T1 and T2 (Figure 1), while in T3, a temperature higher than 46.0°C was recorded at the end of the 30-day composting process. Similarly, temperatures during the initial days of vermicomposting rose and continued until the 45^th^ day in T1 and T2, with a declining trend until the 75^th^ day. Finally, after 75 days, the temperature, irrespective of charcoal addition, reached a near ambient temperature ranging from 26.3-27.0°C as shown in Figure 2.

**Figure 3:**
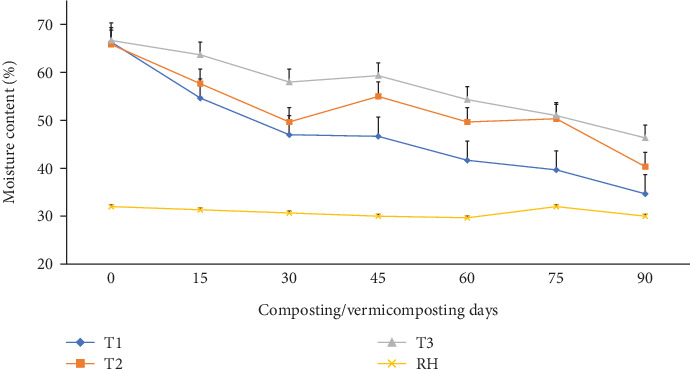
The moisture content changes (%) during the vermicomposting process. T1 = WH + cow manure (2 : 1) + 7.5%charcoal; T2 = WH + cow manure (2 : 1) + 5%charcoal; T3 = WH + cow manure (2 : 1).

The MC measured during the composting and vermicomposting processes is shown in Figure 3. The average MC at the beginning of the composting process was 66.0%, which was significantly (p <0.05) reduced after 30 days of composting to 47.0–49.6% in T1 and T2 and to 58.0% in T3 compared to the initial composting day. No leachate was lost during the 90-day process as the decomposition process was made in a no-leak cement brick wall (Figure 1). MC in all composting bins after 30 days was maintained at a higher level than the room relative humidity (30.6%) (Figure 3). During vermicomposting, MC in T1 and T2 after 30-45 days was significantly (p <0.05) higher than T3 by 45.0%. However, during the 45–90 days, a significantly (p <0.05) higher MC reduction was observed in the order of T1 (52.2%), followed by T2 (50.0%), and T3 (32.5%) compared to the initial values. Considering the entire vermicomposting process, both T1 and T2 exhibited significantly (p <0.05) greater MC reduction than T3 (Figure 3).

**Figure 4:**
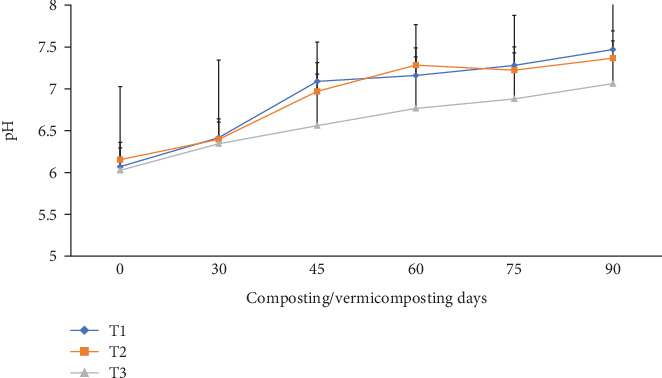
The pH changes during composting and vermicomposting. T1 = WH + cow manure (2 : 1) + 7.5%charcoal; T2 = WH + cow manure (2 : 1) + 5%charcoal; T3 = WH + cow manure (2 : 1).

The pH change was slightly (p <0.05) increased at the end of the composting and vermicomposting processes compared to their initial values in all bins (Figure 4). The initial pH was 6.07, 6.15, and 6.02 before composting, which increased to 6.41, 6.39, and 6.34 at the end of composting and to 7.47, 7.36, and 7.06 after vermicomposting in T1, T2, and T3, respectively. The pH increment was about 23.0% (T1) to 17.2% (T3) after the vermicomposting process compared to the initial value.

**Figure 5:**
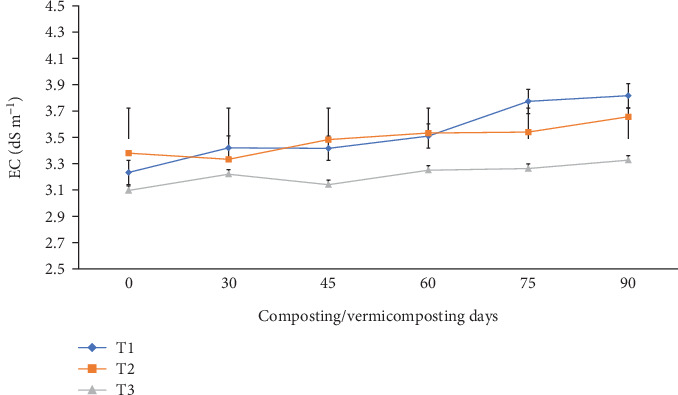
The EC changes during composting and vermicomposting. T1 = WH + cow manure (2 : 1) + 7.5%charcoal; T2 = WH + cow manure (2 : 1) + 5%charcoal; T3 = WH + cow manure (2 : 1).

As shown in Figure 5, the EC varied significantly (p <0.05) in all bins at the end of composting and during vermicomposting. The EC values of the initial feed mixtures were in the range of 3.09–3.23 dSm^−1^, while the final EC of the vermicompost increased to 3.32–3.82 dSm^−1^ in all bins. A greater EC increment was noticed in the order of T1 (18.2%) > T2 (7.99%) > T3 (7.44%) at the end of vermicomposting.

**Figure 6:**
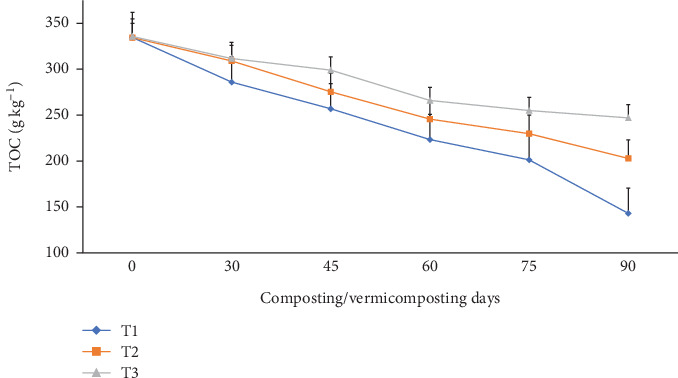
The TOC changes during composting and vermicomposting. T1 = WH + cow manure (2 : 1) + 7.5%charcoal; T2 = WH + cow manure (2 : 1) + 5%charcoal; T3 = WH + cow manure (2 : 1).

The TOC content in all bins significantly (p <0.05) reduced from 334–335 g kg^−1^ to 286–311 g kg^−1^ during composting and further reduced to 142–247 g kg^−1^ at the end of vermicomposting in all bins (Figure 6). Compared to the initial days, the highest TOC reduction was observed in T1 (14.5%), followed by T2 (7.62%) and T3 (7.14%) during composting, and T1 (57.3%), T2 (39.4%), and T3 (26.4%) at the end of vermicomposting (Figure 6).

### 3.3. Changes in Nutrients: *TN, TAP, and TK*

**Figure 7:**
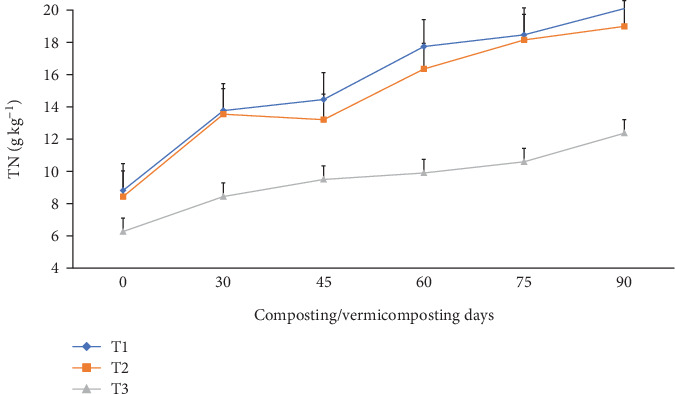
The TN (g kg^−1^) changes (dynamics) during composting and vermicomposting. T1 = WH + cow manure (2 : 1) + 7.5%charcoal; T2 = WH + cow manure (2 : 1) + 5%charcoal; T3 = WH + cow manure (2 : 1).

**Figure 8:**
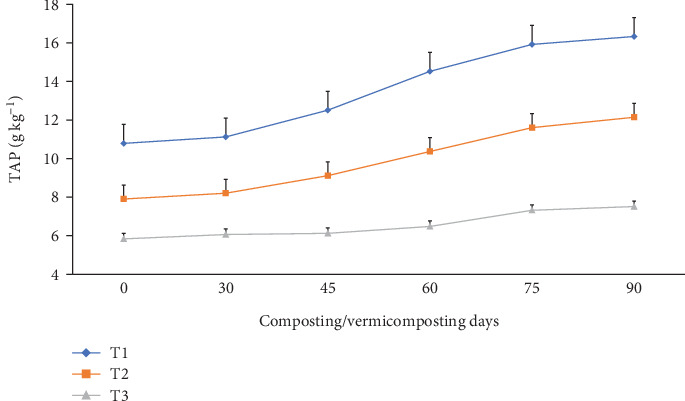
The TAP (g kg^−1^) changes (dynamics) during composting and vermicomposting. T1 = WH + cow manure (2 : 1) + 7.5%charcoal; T2 = WH + cow manure (2 : 1) + 5%charcoal; T3 = WH + cow manure (2 : 1).

**Figure 9:**
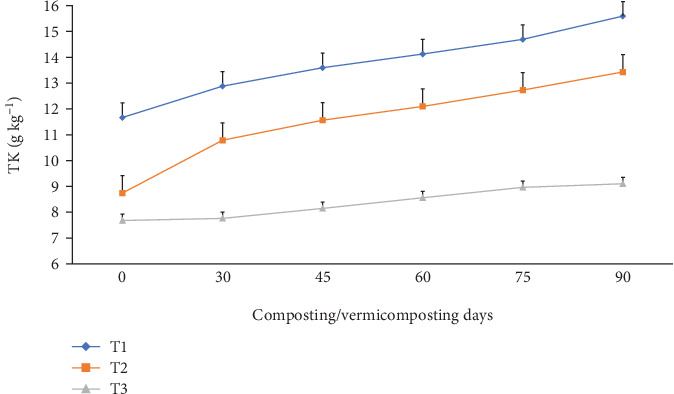
The TK (g kg^−1^) changes (dynamics) during composting and vermicomposting. T1 = WH + cow manure (2 : 1) + 7.5%charcoal; T2 = WH + cow manure (2 : 1) + 5%charcoal; T3 = WH + cow manure (2 : 1).

The TN, TAP, and TK varied significantly (p <0.05) at the end of the composting and vermicomposting processes among all bins (Figures 7, 8, and 9). There was an increment in TN, TAP, and TK throughout the composting and vermicomposting processes. The highest increment in TN, TAP, and TK was observed in T1 and T2, whereas the least was observed in T3. The initial TN was 8.81, 8.37, and 6.26 g kg^−1^ in T1, T2, and T3, respectively. This increased by 56.2, 60.8, and 34.7% during composting and by 128, 125, and 97.4% during vermicomposting in T1, T2, and T3, respectively (Figure 7). The initial TAP was 10.8, 7.90, and 5.84 g kg^−1^ in T1, T2, and T3, respectively (Figure 8). This increased by 3.08, 3.83, and 3.88% during composting and by 51.4, 53.7, and 28.7% during vermicomposting in T1, T2, and T3, respectively. As shown in Figure 9, 11.67, 7.90, and 7.68 g kg^−1^ of TK were recorded in T1, T2, and T3, respectively, during the initial analysis and increased by 10.4, 23.4, and 1.04% at the end of composting and by 33.7, 53.6, and 18.5% at the end of vermicomposting, respectively. The lowest TN, TAP, and TK values were found in T3.

### 3.4. Changes in C/N Ratio

**Figure 10:**
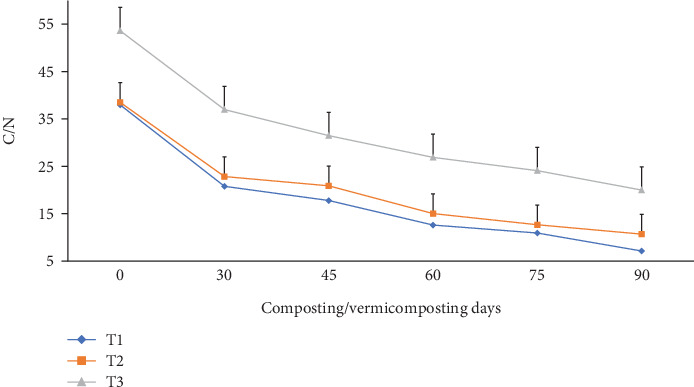
C/N ratio changes during composting and vermicomposting. T1 = WH + cow manure (2 : 1) + 7.5%charcoal; T2 = WH + cow manure (2 : 1) + 5%charcoal; T3 = WH + cow manure (2 : 1).

As shown in Figure 10, the C/N varied significantly (p <0.05) in all bins during the composting and vermicomposting periods. Charcoal-treated bins showed a higher C/N reduction than without. Compared to the initial substrate mixture, the C/N was 45.3, 40.7, and 31.1% lower at the end of composting and further decreased by 81.3, 72.3, and 62.7% at the end of vermicomposting, respectively, at T1, T2, and T3 (Figure 10). The maximum C/N reduction was observed in both T1 and T2, while the lowest C/N was observed in T3.

### 3.5. Growth and Reproduction of Earthworms

**Figure 11:**
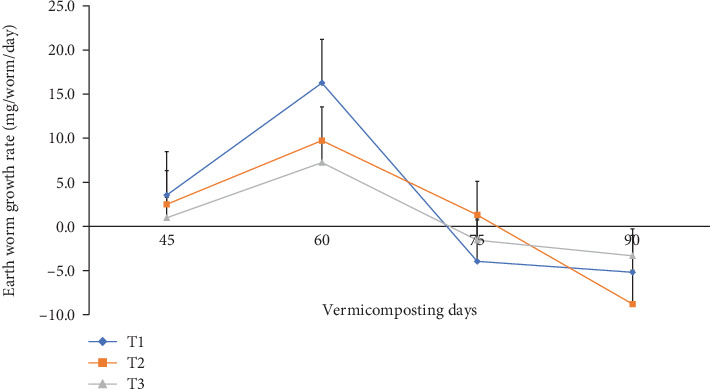
Earthworm growth mass change (mg worm^−1^) during vermicomposting. T1 = WH + cow manure (2 : 1) + 7.5%charcoal; T2 = WH + cow manure (2 : 1) + 5%charcoal; T3 = WH + cow manure (2 : 1).

**Figure 12:**
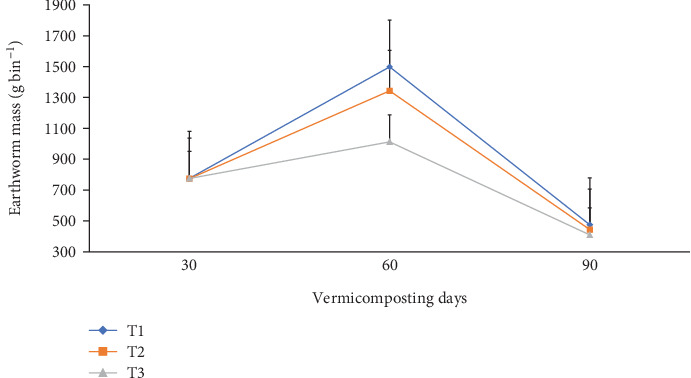
Earthworm mass change (g bin^−1^) during vermicomposting. T1 = WH + cow manure (2 : 1) + 7.5%charcoal; T2 = WH + cow manure (2 : 1) + 5%charcoal; T3 = WH + cow manure (2 : 1).

The total earthworm number, cocoon number, hatchling number, earthworm growth rate (mg worm^−1^), and total earthworm weight (g bin^−1^) during vermicomposting are presented in Figures 11 and 12 and Table 3. After 30 days of worm addition, the total earthworm number significantly (p <0.05) increased from 865 to 1225, 1200, and 1118 with 41.6, 41.2, and 18.0% increments in T1, T2, and T3, respectively. However, the earthworm number declined to 356, 348, and 210 in T1, T2, and T3, respectively, at the end of the vermicomposting (Table 3). The average cocoon number after the first cocoon count was about 266, 262, and 202 in T1, T2, and T3, respectively, which then decreased by 54.3, 57.2, and 59.4% at the end of the vermicomposting. Besides, the hatchling number decreased by 41.7%, 49.3%, and 27.3% in T1, T2, and T3, respectively, at the end of vermicomposting. The average earthworm growth rate showed an increasing trend from 1.00–3.50 to 7.20–16.3 during the 45^th^ to 60^th^ days and declined to 1.60–4.00 during the 60^th^ to 75^th^ days, which further decreased to 3.30–8.80 during the 60^th^ to 75^th^ days of vermicomposting (Figure 11). After 30 days of earthworm addition, the total earthworm weight increased from 775 g per bin to 1498, 1343, and 1012 g but decreased to 475, 443, and 409 g in T1, T2, and T3, respectively (Figure 12).

### 3.6. Vermicompost Mass Change

Charcoal addition prior to composting significantly (p <0.05) reduced the vermicompost mass at the end of the 90 days (Table 4). The initial mass of the substrate mixtures ranged from 18.0–19.3 kg bin^−1^, while the final compost and vermicompost were reduced to 12.2-15.4 and 5.67–7.93 kg bin^−1^ in all bins. The maximum reduction in the compost and VC mass was noticed in T1 (63.0, 71.7%), followed by T2 (69.3, 67.9%) and T3 (85.6, 55.9%) compared to the initial substrate. Significantly, the highest and lowest VC yields were obtained in T3 (44.1%) and T1 (29.3%), respectively, compared to the initial mass.

### 3.7. Effect of VC Extract on Seed Germination, and Seedling Emergence Index

**Figure 13:**
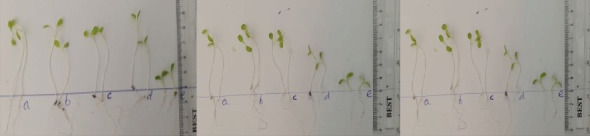
Effect of VC extract type treatment on lettuce seed germination 3 DAS (where a, b, c, d, and e represent 0%, 25%, 50%, 75%, and 100% VC extract concentration, respectively). T1 = WH + cow manure (2 : 1) + 7.5%charcoal; T2 = WH + cow manure (2 : 1) + 5%charcoal; T3 = WH + cow manure (2 : 1).

The results of lettuce germination tests indicated GI values ranged from 55.8–80.4% in T1, T2, and T3 (p <0.05), respectively (Figure 13 and Table 5). Similarly, the GI of lettuce after VC prepared from T1, T2, and T3 in five dilutions (a, b, c, d, and e, 0, 25, 50, 75, and 100%, respectively) significantly (p <0.05) varied in the range of 47.6-100% (Figure 13 and Table 6). At higher VC concentrations, lower lettuce GI values were observed (Figure 13 and Table 6).

## 4. Discussion

### 4.1. Changes in Temperature and Moisture Content

The quick rise in the thermophilic stage (>45°C) within 3 days of composting in T1 and T2 was maintained above 50.0°C for about 17 days, as seen in Figure 1. This rapid increase at the thermophilic stage could be associated with the collective effect of charcoal [54], moisture reduction [55], and rising microbial activity [41, 56]. Therefore, charcoal-treated bins (T1 and T2) experienced a higher rising temperature (Figure 1) linked to the charcoal's higher surface area and porosity [54], which facilitates moisture loss (Figure 3) through evaporation and microbial activity [55], which led to better maturity [39, 56]. Besides, charcoal amendments also promote oxygen transfer and provide a suitable habitat for the rapid establishment of microbial activity that creates a synergistic effect in enzyme activities during the vermicomposting of organic matter [42]. According to [42], the rapid breakdown of readily available organic matter and nitrogenous compounds by microorganisms increases temperature. However, the temperature gradually showed the start of the cooling phase as the complex organic compounds were well-degraded during the mesophilic and thermophilic phases, and simpler compounds were left to be consumed by microorganisms in the last stage [55] as shown in Figure 1.

The composts in charcoal-treated bins (T1 and T2) satisfied the standard sanitary requirements and could kill pathogens and weed seeds because the thermophilic stage was maintained over 45°C for 17 days and hence was less phytotoxic [57]. Also, the declining temperature trend observed during the initial days of vermicomposting (Figure 2) could be attributed to water addition [47] (Figure 3), turning [58], the high surface area, and the porosity of charcoal's suitability for earthworm burrowing [54]. Such a temperature reduction favors mesophilic microbe and earthworm reproduction, growth, and biomass [59] (Figures 12 and 13 and Table 3) and vermicomposting activity [54]. The fall in temperature, irrespective of charcoal addition after 75 days of vermicomposting (Figure 2), might be due to decreased microbial activity as organic material becomes more decomposed and essential nutrients are depleted because the VC is attaining maturity [39, 42, 55]. Also, the constant watering and turning might improve aeration [47], and worm burrowing cools the temperature [49]. Furthermore, the porosity of the substrate promotes aeration during thermophilic composting, creating a more favorable environment for thermophilic bacterial activity and temperature increases [21]. This, in turn, leads to higher water usage and reduced moisture content [60]. To address this issue, the mixtures were regularly watered and turned mixtures [47].

Compared to the initial days, significantly (p <0.05), the highest MC reduction was observed at the end of the 30 days of composting in T1 and T2 (47.0 to 66.0%) than in T3 (58.0 to 66.0%), which fulfills the optimum standard for composting [61]. Such a higher MC reduction during composting could be attributed to moisture loss due to turning [58], the nature of the substrate mixture, charcoal's higher surface area and porosity [54], and the higher thermophilic temperatures (>45°C) associated with heat generated [57] in T1 and T2 that might be consumed by microbes or lost due to evaporation than without charcoal (T3). According to Jain and Kalamdhad [57], moisture and temperature are important factors that govern microorganism activity and the subsequent degradation of organic molecules. The MC in all composting bins after 30 days was maintained higher than the relative humidity, which was in the acceptable range (45.0-65.0%) for composting, irrespective of charcoal amendment. The relatively higher MC in T3 until day 30 could be due to the poor decomposition and porosity of the WH and the increased production of metabolic water by microbial activity during the composting process [57]. After the 45^th^ day, a significantly higher MC reduction was observed in charcoal-treated bins (Figure 3), which could be attributed to the earthworm's borrowing activity that facilitates moisture reduction [62], the rapid microbial decomposition process due to the higher earthworm population that causes elevated heat generation [49, 63], and evaporation loss [40]. The higher final moisture reduction observed towards the end of the maturity phase (75-90^th^ days) in all bins is a key benefit in the vermicomposting process in terms of mass and volume reduction, transport cost, storage, and use of the product thereafter [48, 64].

### 4.2. Changes in pH, EC, and TOC

As illustrated in Figure 4, the pH results in T1 and T2 at the end of the vermicomposting process demonstrated significant pH shifts from acidic to a near-neutral range (7.06-7.47), which is ideal for agricultural application [25, 65]. The quality and palatability of the substrate used in the vermicompost can impact earthworm and microorganism growth rate, reproduction, and activity [40, 66]. Depending on the chemical composition of the substrate used for vermicomposting, a pH may shift in either direction [66]. The *E. fetida* grows best on substrates with a pH between 6 and 8 [25]. Findings in this study showed that charcoal amendment (T1 and T2) decreased the acidity of the vermicomposting mixture, which complements the buffering properties of earthworm external mucus [66]. In the current study, the rise in pH during composting can be attributed to higher aeration that resulted in higher microbial activity and decomposition rates, along with the absorption of ammonia produced by the decomposition of nitrogen-containing organic compounds and degradation of organic acids leading to the release of CO_2_ and a more stable compost [67–69]. In their study Zhang et al. [70] attributed the increase in the pH to the release of a large amount of NH_4_^+^-N from the decomposition of organic matter, such as protein and nitrogen. Conversely, the decomposition of organic compounds into organic acids will result in a decrease in pH [67]. Salt solubility decreases with increasing pH due to anion protonation EC [38].

The significant (p <0.05) EC (salinity) increase in T1 and T2 at the end of vermicomposting compared to T3 (Figure 5) can be attributed to the release of soluble salts, ammonium, and other inorganic ions/compounds during the vermicomposting process [21, 39, 40, 48]. The EC is influenced by raw materials used for vermicomposting [66], microbial activity, minerals and ions generated by earthworms during ingestion and excretion, and temperature generated during vermicomposting [71]. On the other hand, the EC influences the survival and growth of earthworms and plant growth, determining the vermicompost quality [41]. Optimum EC for earthworm growth is <4 dSm^−1^ [72]. Also, optimum EC (<4 dSm^−1^) is ideal for plant growth because it allows organic fertilizers to slowly release mineral salts [72].

Our findings demonstrate that the increased EC from 3.09 to 3.82 dSm^−1^ (Figure 5) in all bins correlated to the growth rate, survival rate, and cocoon production of *E. fetida* decrease in treatment without charcoal, which is consistent with the findings of [72]. In contrast, other reports showed a decrease in EC during the vermicomposting process attributed to the release of organic acids [38], decomposition of organic matter, and the mineralization of compounds by earthworms [73]. Earthworms metabolize and bioaccumulate minerals and metal ions [74] and microbial consumption of salts. As indicated by Mukhopadhyay [38], pH and EC interact in opposite directions; he showed that EC decreased by 81.0%, while pH increased by 21.1% across all treatments over the 7-week period. Findings in the current study showed that the final vermicompost products in all bins are within the optimum range (<4 dS m^−1^), ideal for agricultural applications [36]. Vermicompost application to plants with an optimum EC level supports plant and soil health by allowing organic fertilizers to slowly release mineral salts, improving nutrient uptake and microbial diversity. It also has water use efficiency, requiring less irrigation water than conventional fertilizers in water-scarce areas [75]. Soils treated with an optimum EC range have no soil toxicity and promote seed germination and plant physiology [39, 41].

Because of its extremely porous nature, charcoal greatly improves soil aeration, retains moisture, promotes gas exchange, and provides the perfect environment for bacteria [76] that efficiently decompose organic materials [63, 68, 77]. Its enhanced aeration lowers the possibility of anaerobic situations and lessens toxic byproducts such as nitrogenous chemicals and methane [78]. Its rich microbial population, mainly bacteria, fungi, and protozoa, helps accelerate the decomposition process and improve nutrient cycling to produce higher-quality vermicompost that increases soil fertility and disease resistance [79, 80]. Additionally, charcoal improved aeration benefits earthworms by facilitating their mobility and guaranteeing sufficient oxygen for respiration, both of which promote general health [81].

Additionally, charcoal retains substantial moisture vital for both microbial activity and earthworm growth and survival [82, 83], adsorbs antibiotics, toxic compounds, heavy metals, and pesticides [84, 85]. By mitigating the harmful effects of these toxins, charcoal fosters a favorable environment for beneficial microorganisms, promoting their essential functions like nutrient mineralization [80, 86]. It also retains essential nutrients, prevents leaching, and ensures nutrient and moisture availability to plants [82, 87]. Collectively, these features create a synergistic effect that maximizes composting efficiency, resulting in nutrient-rich vermicompost [80, 88].

In contrast, vermicomposting without charcoal causes the composting mixture density to increase, limits air pockets, and leads to poor gas exchange [88]. Absence of charcoal addition raises the risk of anaerobic conditions, producing harmful byproducts like methane and slowing aerobic microorganisms, which prolongs decomposition and results in incomplete organic matter breakdown [88] and inhibits seed germination [89] and plant health. Overall, the absence of charcoal leads to lower soil fertility, reduced crop yields, and increased susceptibility to pests and diseases [80, 88].

The significantly (p <0.05) highest TOC reduction was observed in charcoal-treated bins, especially since the thermophilic phase lasted throughout the entire process (Figure 6). This was explained by the abundance of carbon in T1 and T2 compared to T3 [90]. These results are in agreement with the findings of Nath and Singh [39, 91], who reported that WH co-vermicomposting with buffalo dung and gram bran (2:1:1) using *E. fetida* decreased TOC (by 28.4%). The highest TOC reduction measured in the charcoal-treated bins could be attributed to the fast OM degradation by the collective action of earthworms and microorganisms as a source of energy for the worms and microorganisms and carbon assimilation due to the respiration of microorganisms and earthworms [40, 46, 55]. While the absence of charcoal in the control bin slowed the degradation process, which is accompanied by a low TOC reduction trend [37, 39, 46]. The present TOC reduction in the final vermicompost result shows in conformity with earlier findings of Das and Deka [39] and Das et al. [40], which they attributed to the carbon mineralization, humification, and decrease of mass in the substrate materials during composting and vermicomposting. Besides, the joint activity of earthworms and microbes consumes significantly higher organic carbon as a source of energy and leads to TOC reduction during the vermicomposting systems [39]. Moreover, the decrease in TOC during vermicomposting systems by Das et al. [40] was attributed to earthworm mucus and gut enzymes accelerating the degradation of organic matter in the substrate mixture and organic carbon loss in the form of CO_2_, which causes a substantial decrease in TOC in the final products. In fact, the lower TOC level at the end of vermicomposting indicates the maturity and phenolic compounds [39].

### 4.3. Changes in TN, TAP, and TK

The TN, TAP, and TK increased significantly (p <0.05) at the end of composting and vermicomposting in all bins (Figures 7, 8, and 9). Despite the high temperatures recorded (above 55°C) during the thermophilic phase for both T1 and T2, which could evidently experience volatilization and a consequent loss of TN, TAP, and TK, these values showed an increasing trend till the end of the process [39, 45, 57]. The TN, TAP, and TK increment results observed during composting and vermicomposting were also reported [39, 45, 91]. According to Nath and Singh [91], vermicomposting WH with buffalo dung and gram bran (2:1:1) using *E. fetida* increased the TN by 198% and the TP by 19.1%. In another study by Ronghua et al. [92], charcoal addition to pig manure and rice husk aerobic composting systems improved microbial decomposition and reduced nutrient leaching. [41], discussed that bulking agent addition to vermicomposting of patchouli bagasse with cow manure by *E. fetida* effectively reduced the bioavailability and leachability of nutrients, which are useful for soil health and plant growth. The higher TN levels in charcoal-containing bins (T1 and T2) (Figure 7) could be attributed to the mineralization action of microorganisms and earthworms during digestion, nitrogenous excretory products, mucus, body fluid, enzymes, and, furthermore, the decaying of dead tissues of worms and microbes during vermicomposting [37]. The highest TN enhancement in charcoal-treated bins in comparison to untreated bins agrees with other earlier works [39, 42]. These authors have discussed that vermicomposting with additive substrates significantly increased the microorganism population and diversity manyfold.

In another study by Ronghua et al. [92], a 10.0% charcoal addition to pig manure composting for 60 days promoted organic material decomposition, accelerated compost detoxification, increased TN by 20.5% to 53.0%, and increased the germination rate of *D. pluvialis* seeds by 17.6% to 41.2%, and the germination index reached 1.02 to 1.44. This might be due to the fact that charcoal in a composting system supplies adequate air space for aeration [63]. The TN, TAP, and TK nutrient enhancements in T1 and T2 during the composting-vermicomposting processes might be attributed to the presence of a suitable environment for the microbial decomposition to release the organically bound nutrients into available forms compared to T3 [25, 36, 66].

On the other hand, the lowest TN enhancement in T3 could be linked to the slow substrate degradation rate associated with the poor porosity of WH, which retains more water and causes worm mortality or inhibits earthworms' growth and reproduction [26]^,^ compared to the charcoal-treated bins. Charcoal is one of the most common low-cost residues of carbonized products [54], which improves the earthworm feeding ability and hence improves TN, TAP, and TK nutrients in the WH vermicompost [81]. The other reason could be that WH releases allelochemicals that have growth inhibition effects for the earthworms, due to which less TN was produced in T3 compared to the other [4], such as nitrogenous waste excretory substances like mucus, enzymes, and hormones, due to which less TN was produced in T3 compared to the other bins. A similar TAP-increasing trend has also been observed in earlier studies [93]. Yadav and Garg [36] have reported a 29.5% to 75% TAP increment in the vermicompost produced from biogas plant slurry and parthenium weed mixture. The increase in TAP (Figure 8) could be attributed to the joint action of earthworm gut enzymes and the high rate of organic matter mineralization [94] which unlocks the bonded phosphorous and potassium that are bound to the higher molecules [95] in charcoal-treated bins compared to those without. Hanc and Chadimova [64], in their study on nutrient recovery from apple pomace waste, reported a maximum TAP increase of 11% at the end of vermicomposting. The increase in TK (Figure 9) in charcoal-amended bins could be attributed to the highest earthworm growth (Figures 11 and 12 and Table 3) and hence enhanced mineralization. The increase in N, P, and K nutrient availability in the vermicompost samples compared with the initial substrate has also been reported by the previous workers [39, 40]. In their study, Ferraz Ramos et al. [48] and Das and Deka [39] related the increase in N, P, and K nutrient availability at the end of vermicomposting compared with the initial substrate to substrate used, rate of organic matter degradation and nutrient mineralization, and a decrease in substrate volume (due to concentration) [34, 96]. Furthermore, Das and Deka [39] and Das et al. [40] noted that a higher nitrogen content in the final vermicompost is associated with the release of carbon dioxide and water loss due to the rapid mineralization of organic matter. This process involves the release of excretory materials, earthworm mucus, body fluids, enzymes, decomposing tissues, and carbon-rich mucus, along with hormones and enzymes throughout vermicomposting. The enhanced level of AP in the vermicomposted materials, according to Das et al. [40] and Kumar Badhwar et al. [97], was attributed to substrate type, the joint action of earthworms and microbes, which accelerated the enhancement of AP in the vermicompost samples, the actions of phosphate-solubilizing bacteria, and the release of fecal phosphatase enzyme by earthworm gut microorganisms. Besides, the higher TP levels seen during composting may be attributed to phosphorus mineralization, bacterial consumption, organic matter decomposition, and mass loss [21]. Similarly, Das and Deka [39] explained the rise in TK content in the vermicompost samples to the synergistic activities of earthworms and microorganisms that may increase the mineralization of substrates.

Charcoal stabilizes nitrogen by adsorbing ammonium and nitrate [92], prevents leaching, and promotes plant uptake. Its adsorption properties also improve phosphorus availability by binding it in a way that makes it more accessible to plants [82]. Increased microbial activity from charcoal fosters the release of organic acids, which solubilize bound phosphorus, thereby enhancing total available phosphorus (TAP) levels [80, 88]. While potassium is typically more soluble and less prone to fixation, charcoal increases its retention and prevents loss through leachate within the compost [87]. As microbial populations grow, they help solubilize potassium from organic matter and increase its availability [98]. The simultaneous increase in TN, TAP, and TK due to charcoal can be attributed to the enhanced biological activity in the composting process [80]. The interconnected TN, TAP, and TK increase stems from common processes like mineralization, which breaks down organic matter and releases nutrients influenced by microbial actions and charcoal interactions [80, 88].

### 4.4. Changes in C/N

The C/N was significantly reduced (p <0.05) at the end of composting and less at the end of vermicomposting in all bins (Figure 10), which is consistent with earlier works [46, 55, 61, 82]. According to Nour El Houda Chaher [55], once the biodegradability of the OM started, the C/N ratio of the mixtures started decreasing due to the microorganism development in the composting and the combined action of worms and microflora during the vermicomposting process. The highest C/N ratio reduction was observed in T1, followed by T2 and T3 during the entire composting process. The C/N ratio reduction results at the end of vermicomposting are also supported by the findings of earlier works [27, 55, 61] due to organic C loss as CO_2_ through microbial respiration loss and TN gain during the decomposition process. As stated by Bernal et al. [27], a 47.2%. C/N ratio reduction was observed at the end of 110 vermicomposting days.

The C/N reduction in the final product explains the stability and maturity of the vermicompost [61]. A stabilized and mature vermicompost resists further organic matter microbial decomposition, indicating the absence of biodegradable material, including several phytotoxic substances showing its suitability for agricultural use for plant growth, and has a biostimulant property [46]. A vermicompost with a final C/N below 15 reflects a satisfactory degree of maturity of organic wastes suitable for agriculture [55, 61]. The reason for the low final C/N in T1 and T2 could be due to the enhanced synergetic effect of earthworms and microbial action in charcoal amendments [82]. The initial C/N of the substrate explains the nutrition quality for earthworms and microbial growth since both carbon and nitrogen are required for an organism's metabolism to function [55]. The initial C/N in T1, T2, and T3 (49.7–69.2) was higher than the optimum range for agricultural use [39]. This suggested that the bioconversion efficiency is largely associated with the initial carbon and nitrogen in the feed mixtures used for vermicomposting.

As reported by other researchers [39, 48], the decrease in TOC and the subsequent increase in TN values during the vermicomposting process have led to lower C/N values in the vermicomposted substrate. As explained by Das and Deka [39], a larger decrease in C/N values in the final products is facilitated by the increased amount of nitrogen and organic matter breakdown throughout the vermicomposting process. This could be attributed to the fact that carbon and nitrogen mineralization results in lower C/N values of the vermicompost. Further, according to Ganguly et al. [99], reductions in C/N levels in vermicomposted substrate may also result from the breakdown of hemicelluloses, cellulose, and other organic materials during the vermicomposting process and carbon absorption into earthworm biomass. The C/N ratio is an important parameter for microbial activity and the worm's fecundity during vermicomposting and a key parameter for compost maturity determination [21, 39]. As stated by Das and Deka biofertilizer [21]. and Pottipati et al. [39], the C/N value below 20 indicates the stabilization of vermicomposted materials, whereas a value of less than 15 confirms their suitability for agronomic use. The results indicate that charcoal addition was effective in managing water hyacinth biomass in vermicomposting using *E. fetida* [21].

### 4.5. Changes in Earthworm Growth and Reproduction

The results of the present study revealed a significantly (p <0.05) higher earthworm number, individual earthworm weight, and total initial earthworm weight in T1, followed by T2, during the 30–60^th^ vermicomposting days (Figures 11 and 12 and Table 3). However, the cocoon number and the number of hatchling juveniles significantly declined at the end of vermicomposting (Table 3). The lower worm dynamics during the 30–45^th^ days and towards the end of vermicomposting could be attributed to the environmental adaptation of the earthworms [45, 48] and lack of available food for the worms and microbes [40, 48], respectively. According to Das et al. [40] and Ferraz Ramos et al. [48], the highest earthworm population increment was observed in the middle of the vermicomposting process (60 days), which they attributed to the worm biomass gained due to the availability of feed and intense substrate degradation by earthworms and microbes, which passed through a higher thermophilic temperature. Ferraz Ramos et al. [48] further explained the high earthworm activity, juvenile number, reproduction rate, earthworm biomass, and worm growth rate in the initial phase of vermicomposting but a reduction trend towards the end of vermicomposting due to low availability of fresh residues and an increase in recalcitrant organic matter.

In a similar study by Malińska et al. [100], sewage sludge–derived biochar before composting municipal sewage sludge and wood chip mixtures resulted in a significantly improved *E. fetida* cocoon number by 213% on Week 4, and the number of juveniles increased 11-fold on Week 6 compared to without biochar. However, the earthworm number and weight reduction towards the end of the vermicomposting phase could be attributed to the low availability of food and organic matter since earthworms could utilize resources for reproduction, shared with newly formed hatchlings and adult earthworms, and also cause an increase in cocoon production [48]. The findings of the present study are consistent with the results of [82] that the application of 1-5% charcoal increased pH from acidic to nearly neutral pH values, consequently improving earthworm activity.

### 4.6. Vermicompost Productivity

Charcoal amendment promoted thermophilic microbial-induced biomass degradation and hence increased thermophilic temperature (Figure 1) decomposition and contributed to high mass reduction (Table 4), biomass stabilization, and pathogen reduction [68]. The high mass change found in the current study was consistent with Światek et al. [101], who reported that vermicomposting food industry sewage sludge with 5% biochar produced from waste woodchips resulted in no earthworm death at the end of 56 vermicomposting days. The high mass reduction from charcoal-amended bins (T1 and T2) could be attributed to the increased earthworm activity that enhanced the vermicomposting process, which in turn led to the highest mineralization rate and vermicompost quality compared to without. VC productivity is influenced by the decomposition process facilitated by charcoal addition, frequent watering, and turning, which in turn promote both microbes and earthworm activity. The VC productivity found in the current study was consistent with that reported by Hanc and Chadimova [64], who have reported that vermicomposting apple pomace waste with straw produced about 35% weight and 15% volume of the feedstock, respectively. Similarly, Zziwa et al. [102] in their report showed a 57–65% mass reduction after a 60-day vermicomposting of pineapple peels and cattle slurry (3:1 w/w) in a batch system using *Eudrilus euginae*.

### 4.7. Effect of VC on Lettuce Seed Germination and Seedling Emergence Index

The germination percent (GP) and germination index (GI) of T1, T2, and T3 vermicompost mixtures prepared with five dilutions are presented in Figure 13 and Tables 5 and 6. Results revealed that there are varying germination trends in each extract. The high germination index results of VC (T1 and T2) have been explained by the maturity and stability of the product, which is enriched with hormone-like stimulatory substances [103]. The stimulatory effects of VC extracts on lettuce seed germination and seedling growth were recorded at lower dilutions (Figure 13, where a, b, and c represent 0%, 25%, and 50%, respectively, and Tables 5 and 6) that improved GI but were inhibited at higher dilutions (Figure 13, where d and e represent 75% and 100%, respectively, and Tables 5 and 6). Matured VC is considered a stabilized product and a source of organic fertilizers and growth-promoting substances that influence seed germination [25, 40, 41] and plant root length [41]. According to [41] and Das et al. [40], where vermicompost accelerates tomato and radish seed germination, plant growth, and development as the toxic substances are completely removed by earthworms during the vermicomposting of the substrate mixture. On the other hand, the declining seed germination and growth recorded in the mixture with >50% VC extract concentration could be attributed to the alkaline nature of the substrates coupled with high EC and high salinity [25, 39]. Higher VC extract percentages lead to root growth inhibition due to increased alkalinity, nutrients, and metals during vermicomposting. The near-neutral nature of extracts from T1 and T2 better suits early seedling development [55]. In fact, the GI indicates that more than 80% of biomass-based products are safe to apply to the soil [21].

## 5. Practical Application of This Study

The combined composting-vermicomposting process is a zero-waste management practice with low environmental impacts. Given the year-round huge volume of WH biomass availability, it would be ideal for the onsite practicality of WH weed management through vermicomposting at low cost with minimum skill. Thus, to be safe, the WH can be collected before flowering, sundried for a couple of days, well chopped, and finely powdered. Finally, the powdered WH can be mixed with a proper amount of cow manure as a source of nitrogen for the earthworms, and microbes and charcoal undergo regular monitoring and management in order to help the earthworm population grow and reproduce. In this study site, charcoal can be easily available in the local charcoal retailers and leftover fine powder unfit for cooking in the household. Earthworms in the bins require 50–65% moisture and ventilation without exposure to direct sunlight. A matured VC can be prepared within or less than 90 days and can be directly applied to agricultural soil since it fulfills quality standards. However, irrespective of the WH's widespread invasion and huge voluminous biomass direct disposal on the lakeside and on the farmers land, extraordinary water content, leachate flow, eutrophication, and seed germination risks require attention. Further, in order to produce cost-effective vermicomposting, socio-economic issues, especially farmers' skills, knowledge, and perceptions towards vermicomposting practices, need to be well addressed.

## 6. Conclusion

This study showed that the use of WH for composting, followed by vermicomposting with cow manure and charcoal amendment, produces a valuable end product. The maturity and stability of the product are explained by a decreased MC, C/N, TOC, phytotoxicity, and malodor with an increased pH, EC, TN, TAP, and TK nutrients that are within the recommended limit for agricultural application. Also, better enhanced earthworm number, weight, hatchlings, and cocoon number in the absence of worm death with the charcoal amendment signified that 5.00–7.50% charcoal created a suitable environment for earthworm survival and activity. The highest VC productivity (32.5%) was recorded due to the 7.5% charcoal amendment. Besides, the highest GI and GP for T1 and T2 explained that the application of charcoal (5.00–7.50%) with WH and cow manure increases the nutrient content and reduces the level of phytotoxicity in the lettuce seed germination index, indicating that charcoal is an ideal substrate mixture for the WH vermicomposting process. The current findings are evident for integrating WH management and sustainable agricultural management and policy strategies in areas suffering from invasive aquatic weeds and agricultural nutrient deficiencies; policymakers ought to think about encouraging the incorporation of water hyacinth and charcoal into organic waste management initiatives. Educating farmers on how to produce quality vermicompost and the advantages of composting could help improve crop yields, promote sustainable agricultural practices, and improve the health of soil and fresh water ecosystems. Eco-friendly waste management and environmental protection may result from the wider adoption of eco-friendly methods that are accompanied by financial incentives. Overall, the resulting vermicompost is an important part of future sustainability projects as it addresses substantial organic waste management with improved agricultural outcomes.

## Figures and Tables

**Table 1:** Composting treatment mixtures.

**Treatment**	**Substrate mixture**
T1	Water hyacinth (12 kg) + cow manure (6 kg) + charcoal (1.35 kg =7.5%)
T2	Water hyacinth (12 kg) + cow manure (6 kg) + charcoal (0.9 kg =5%)
T3	Water hyacinth (18 kg) + cow manure (6 kg)

**Table 2:** Initial physicochemical characteristics of WH, cow manure, and charcoal.

**Treatments**	**PH**	**EC (dS m** ^**−1**^ **)**	**TOC (g kg** ^**-1**^ **)**	**TN (g kg** ^**-1**^ **)**	**C/N**	**TAP (g kg** ^**-1**^ **)**	**TK** **(g kg** ^**-1**^ **)**	**MC (%)**
WH	8.56 ± 0.04	4.52 ± 0.08	336 ± 0.81	7.87 ± 0.04	42.7 ± 0.14	5.48 ± 0.03	9.45 ± 0.08	16.2 ± 0.42
Cow Manure	9.16 ± 0.05	6.51 ± 0.01	269 ± 1.02	9.49 ± 0.03	28.4 ± 0.26	4.43 ± 0.08	4.75 ± 0.07	74.7 ± 0.61
Charcoal	8.52 ± 0.12	3.51 ± 0.04	290 ± 2.05	2.96 ± 0.09	97.9 ± 21.8	10.2 ± 0.08	96.7 ± 2.16	7.63 ± 0.12

Abbreviations: Ash = ash content, C:N = carbon-to-nitrogen ratio, EC = electrical conductivity, MC = moisture content, TAP = total available phosphorus, TK = total potassium, TN = total nitrogen, TOC = total organic carbon, WH = water hyacinth.

**Table 3:** Earthworm, cocoon, and hatchling number.

**Treatment**	**Worm number**			**Cocoon number**		**Hatchling number**	
	**Initial**	**After 30 days**	**After 90** days	**After 30 days**	**After 60** days	**30 days**	**After 60 days**
T1	864 ± 9.70^a^	1225 ± 18.0^a^	357 ± 17.0^a^	267 ± 10.0^a^	122 ± 2.00^a^	709 ± 5.35^a^	413 ± 3.56^a^
T2	864 ± 9.70^a^	1200 ± 11.0^a^	348 ± 2.36^a^	263 ± 5.40^a^	112 ± 2.05^a^	685 ± 6.46^a^	347 ± 5.44^b^
T3	863 ± 9.30^a^	1016 ± 6.24^b^	211 ± 0.94^a^	202 ± 2.16^b^	82.0 ± 2.16^c^	320 ± 0.82^c^	233 ± 5.25^c^

*Note:* Means in the same column followed by the same letter are not significantly different at *p* < 0.05. T1 = WH + cow manure (2:1) + 7.5% charcoal, T2 = WH + cow manure (2 : 1) + 5% charcoal, T3 = WH + cow manure (2 : 1).

**Table 4:** Vermicompost mass change during composting and vermicomposting.

**Treatment**	**Initial mass (kg bin** ^**-1**^ **)**	**Mass at the end of composting (kg bin** ^**-1**^ **)**	**Mass change at the end of composting (%)**	**Mass change at the end of vermicomposting (kg bin-1)**	**Mass change at the end of vermicomposting (%)**
T1	19.3 ± 0.00^a^	12.2 ± 0.08^c^	63.0 ± 0.04^c^	5.67 ± 0.05^c^	29.3 ± 0.24^c^
T2	19.3 ± 0.00^a^	13.4 ± 0.21^b^	69.3 ± 1.12^b^	6.20 ± 0.08^b^	32.1 ± 0.42^b^
T3	18.0 ± 0.00^a^	15.4 ± 0.08^a^	85.6 ± 0.45^a^	7.93 ± 0.13^a^	44.1 ± 0.70^a^

*Note:* Means in the same column followed by the same letter are not significantly different at *p* < 0.05. T1 = WH + cow manure (2 : 1) + 7.5% charcoal, T2 = WH + cow manure (2 : 1) + 5% charcoal, T3 = WH + cow manure (2 : 1).

**Table 5:** Effect of VC extract type on lettuce seed GP and GI.

**Treatments**	**Sl (cm)**	**Rl (cm)**	**GP (%)**	**GI (%)**	**GI classification**⁣^∗^
T1	5.00 ± 0.05^a^	2.53 ± 0.15^a^	93.5 ± 4.79^a^	80.4 ± 0.06^a^	Not phytotoxic
T2	3.80 ± 0.05^b^	2.40 ± 0.13^b^	92.4 ± 4.52^a^	76.2 ± 0.03^a^	Not phytotoxic
T3	2.60 ± 0.04^c^	2.20 ± 0.13^c^	73.8 ± 4.42^b^	55.9 ± 0.07^b^	Strongly phytotoxic

*Note:* Means in the same column followed by the same letter are not significantly different at *p* < 0.05. T1 = WH + cow manure (2 : 1) + 7.5% charcoal, T2 = WH + cow manure (2 : 1) + 5% charcoal, T3 = WH + cow manure (2 : 1).

Abbreviations: GI = germination index, GP = germination percent, Rl = root length, Sl = shoot length.

⁣^∗^[52].

**Table 6:** Effect of VC extract concentration on lettuce seed GI.

**VC extract concentration**	**Sl (cm)**	**Rl (cm)**	**GP (%)**	**GI (%)**	**GI classification**⁣^∗^
0	5.00 ± 0.05^a^	3.00 ± 0.14^a^	100 ± 4.55^a^	100 ± 0.05^a^	Not phytotoxic
25	4.00 ± 0.04^b^	2.22 ± 0.15^c^	78.1 ± 4.18^b^	58.2 ± 0.06^b^	Mild phytotoxic
50	4.00 ± 0.06^b^	2.67 ± 0.13^b^	104 ± 4.33^a^	95.3 ± 0.05^a^	Not phytotoxic
75	3.33 ± 0.03^c^	2.00 ± 0.14^d^	79.6 ± 4.39^b^	53.0 ± 0.04^bc^	Strongly phytotoxic
100	2.67 ± 0.05^d^	2.00 ± 0.17^d^	71.5 ± 4.62^c^	47.7 ± 0.05^c^	<0.4, severe phytotoxic

*Note:* Means in the same column followed by the same letter are not significantly different at *p* < 0.05. T1 = WH + cow manure (2 : 1) + 7.5% charcoal, T2 = WH + cow manure (2 : 1) + 5% charcoal, T3 = WH + cow manure (2 : 1).

Abbreviations: GI = germination index, GP = germination percent, Rl = root length, Sl = shoot length.

⁣^∗^[53].

## Data Availability

All data used in this study will be available up on a prior request from the corresponding author.
