# Supplementary figures and images for: Effect of Charcoal on the Quality of Vermicompost Produced With Water Hyacinth and Cow Manure
Source: ScientificWorldJournal. 2025 Mar 24;2025:1086347. doi: 10.1155/tswj/1086347 (PMC11957873; doi:10.1155/tswj/1086347)

## Slide 1
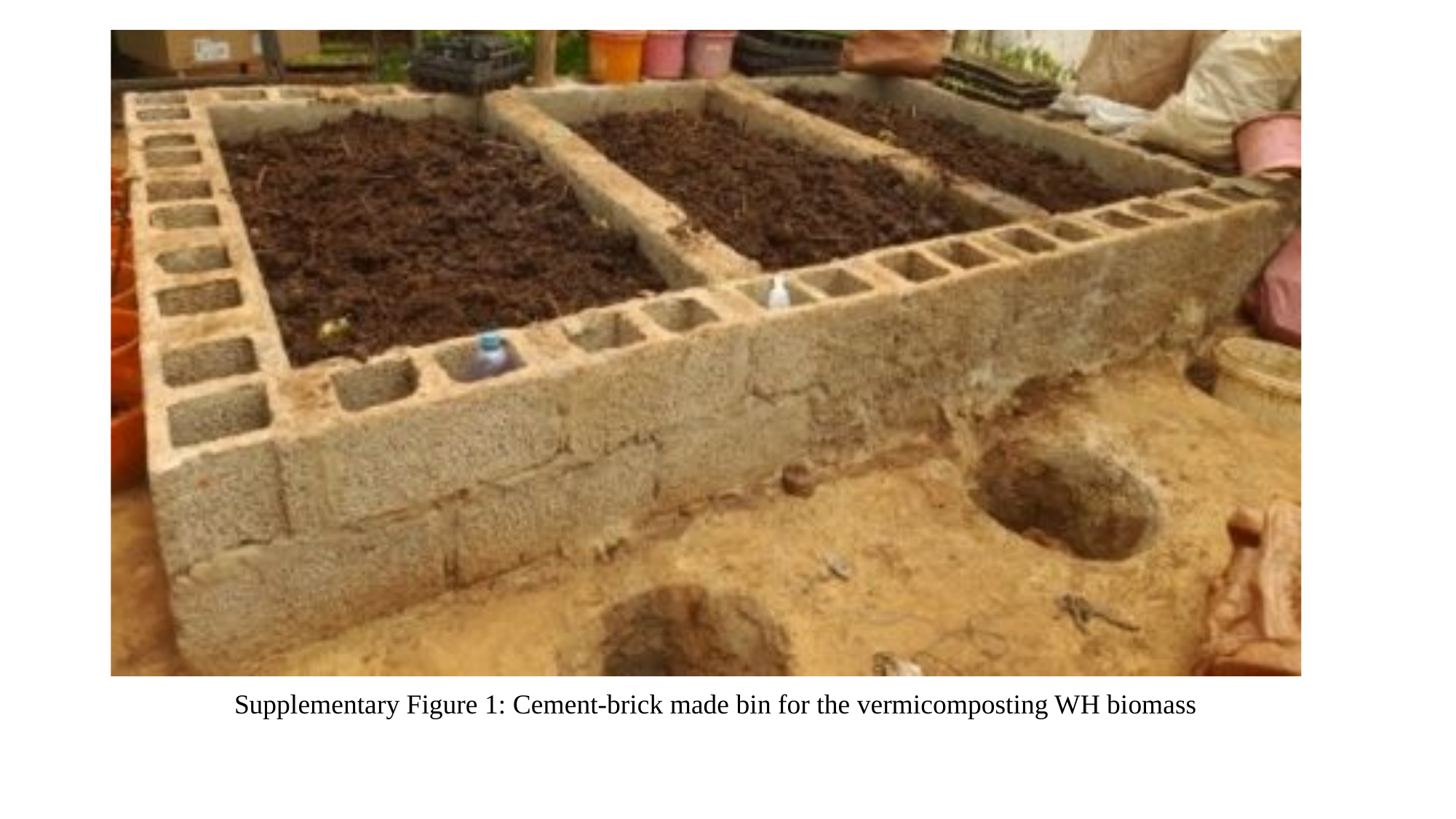

# .
Supplementary Figure 1: Cement-brick made bin for the vermicomposting WH biomass

Supplement: Supporting Information — Additional supporting information can be found online in the Supporting Information section. Figure S1: Cement-brick made bin for the vermicomposting WH biomass. [file 1086347.f1.pptx]
